# Nectar‐Feeding Behavior in the Mallee Ringneck, *Barnardius zonarius barnardi*

**DOI:** 10.1002/ece3.70674

**Published:** 2025-01-23

**Authors:** Amanda E. Hewes, Alejandro Rico‐Guevara, Todd J. McWhorter

**Affiliations:** ^1^ Department of Biology University of Washington Seattle Washington USA; ^2^ Burke Museum of Natural History and Culture Seattle Washington USA; ^3^ School of Animal & Veterinary Sciences University of Adelaide Roseworthy South Australia Australia

**Keywords:** Australia, ecology, feeding, nectarivore, parrot

## Abstract

Opportunistic nectarivory occurs in many avian lineages around the world. In order to understand the implications of this behavior to plant reproduction via pollination and to other nectarivores via competition, more thorough descriptions of opportunistic nectar‐feeding behavior are necessary. We observed nectar feeding of the mallee ringneck, *Barnardius zonarius barnardi* , on flowers of the spotted emu bush, *Eremophila maculata*, in the temperate mallee of South Australia. Here, we describe the nectar‐feeding behavior of *B. zonarius barnardi* and discuss the implications for competition with honeyeaters and the reproduction of *E. maculata* . We also compare the morphology of the feeding apparatus of *B. zonarius barnardi* with that of nectarivorous parrots, lorikeets and lories, to determine whether they share convergent morphological features to facilitate the consumption of nectar. Finally, we suggest avenues for future natural history work to better document opportunistic avian nectarivory in Australian ecosystems.

## Graphical abstract

Mallee Ringneck, *Barnardius zonarius barnardi* , feeding on a flower of *Eremophila maculata* at Gluepot Reserve in South Australia. *B. zonarius barnardi* is an opportunistic nectarivore that uses its unspecialized tongue (red arrow) to remove the nectar from *E. maculata* flowers by making an incision with the lower bill tip and repeatedly probing the incision with the tongue, all while holding the flower between the bill tips.

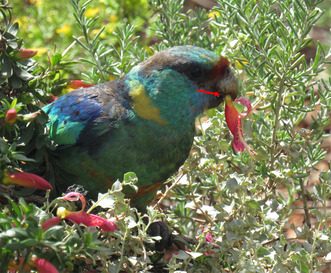

## 1 Introduction

There are approximately one hundred species of parrots known to visit flowers (Ollerton 2024), and throughout Australia, lorikeets, lories, and swift parrots are arguably the most emblematic nectar‐feeding parrots. Lorikeets in particular are brightly colored, gregarious, and can be cacophonous when congregating in a large tree or shrub (Joseph, Merwin, and Smith 2020; Smith et al. 2020). The Mallee Ringneck, *Barnardius zonarius barnardi* , is found within the Platycercinae, or the Australian broad‐tailed parrots (Joseph and Wilke 2006; Schweizer, Guntert, and Hertwig 2012). Within the Platycercinae, *Barnardius* is a member of the core Platycercini (*sensu* Schweizer, Guntert, and Hertwig 2012) a clade that also contains the rosellas (genus *Platycercus*) and the mulga parrot, *Psephotellus varius* (Schweizer, Guntert, and Hertwig 2012). *Barnardius zonarius barnardi* is part of the *Barnardius zonarius* species complex, which occurs throughout inland and western Australia and contains a disputed number of subspecies (Joseph and Wilke 2006). Of the putative subspecies in the *B. zonarius* complex, subsp. *barnardi* is the most distinct morphologically, having a unique combination of plumage colors across the crown, occipital collar, nuchal collar, and frons (Joseph and Wilke 2006). Platycerine parrots are often considered granivores, but the genus *Barnardius* is quite omnivorous, often consuming fruits, berries, vegetal tissue, insects, and flowers in addition to seeds (Forshaw 1973; Juniper and Parr 1998; Higgins 1999; Koutsos, Matson, and Klasing 2001; Menkhorst et al. 2017; Paton 1986). Here, we describe the nectar‐feeding behavior of *B. zonarius barnardi* at flowers of *Eremophila maculata* (F. Muell. 1859), known commonly as the spotted emu bush, and describe the method of nectar extraction. We also discuss the evolutionary relevance of this observation in the context of other nectarivorous parrots, and the ecological relevance to the nectar landscape and potential interactions with honeyeaters (Aves, Meliphagidae), the dominant avian nectarivores in the region.

## 2 Nectar Feeding in *Barnardius zonarius barnardi*

### 2.1 Description of Feeding Behavior

**FIGURE 1:**
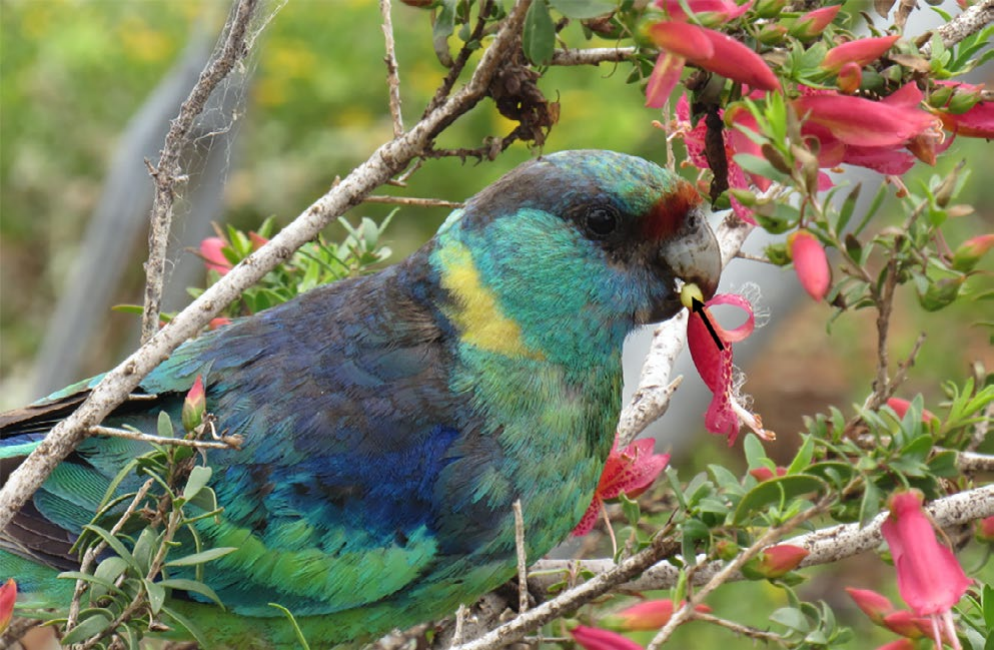
*Barnardius zonarius barnardi* using the bill to orient an *E. maculata* flower. The upper inside surface of the upper bill is used to brace the flower, while the tip of the lower bill is used to pierce it. Arrow indicates floral nectary. Photograph taken by A.E.H. at Gluepot Reserve, South Australia.

**FIGURE 2:**
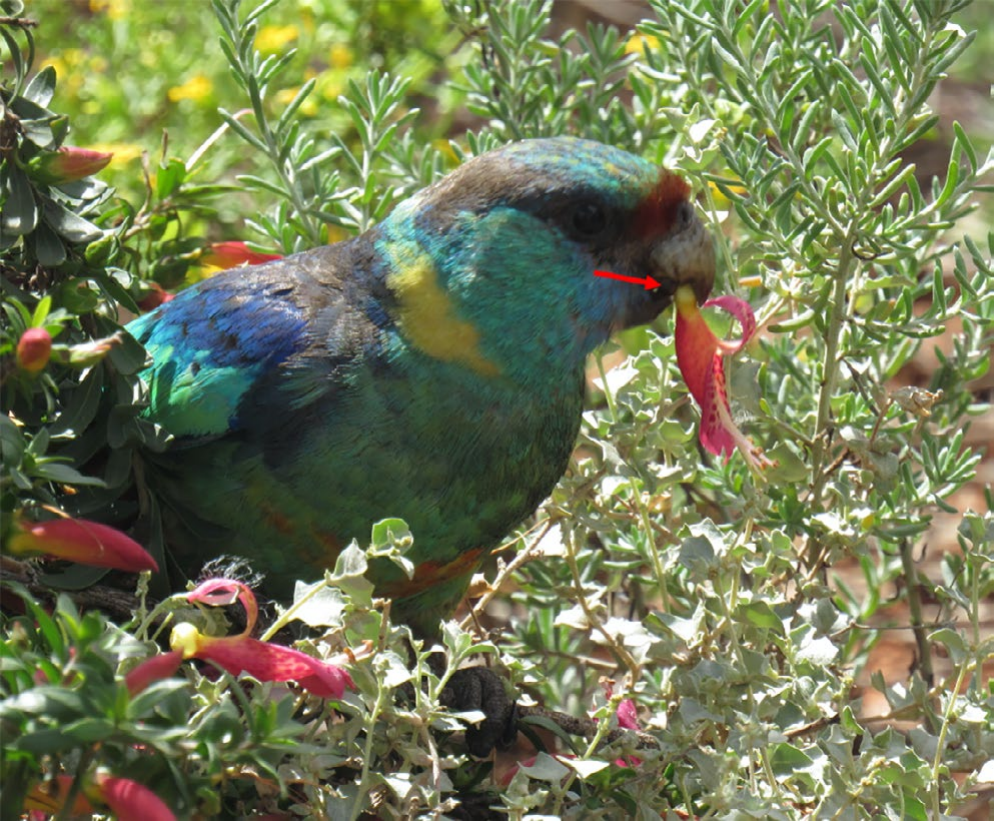
*Barnardius zonarius barnardi* using the tongue (red arrow) to remove nectar from an *E. maculata* flower. Photograph taken by A.E.H. at Gluepot Reserve, South Australia.

**FIGURE 3:**
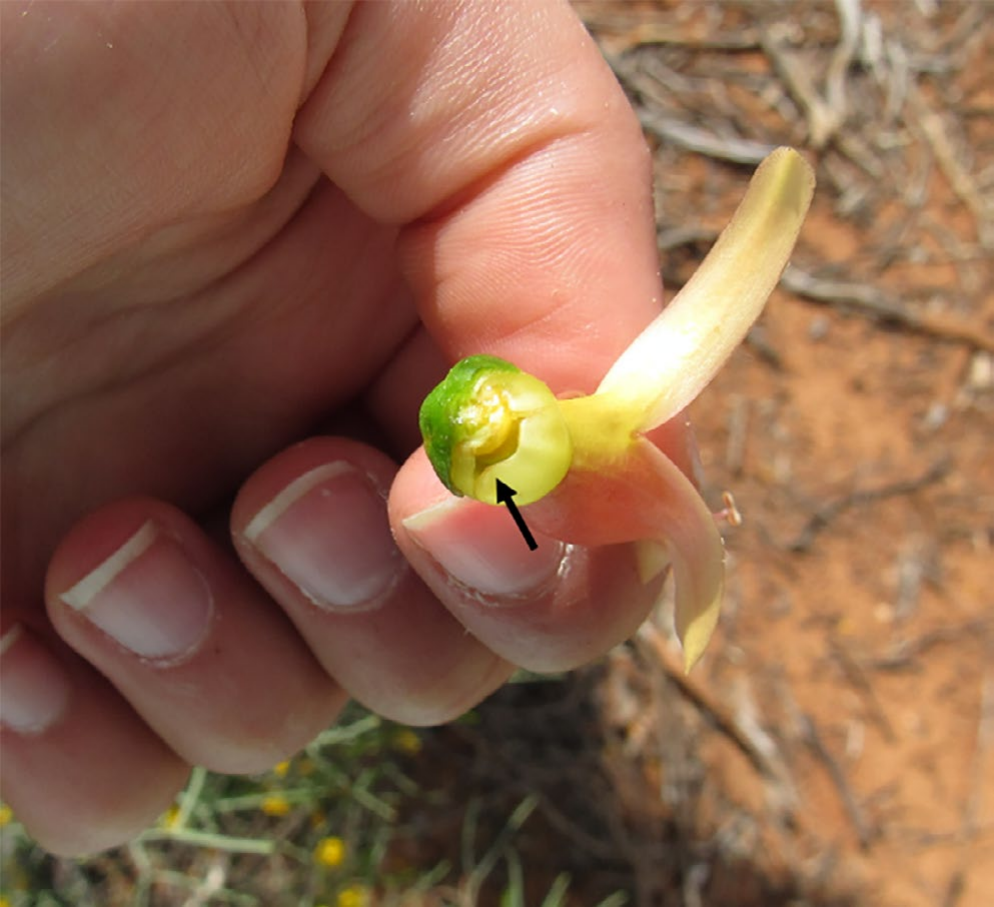
Hole left in base of an *E. maculata* flower after being pierced by a *Barnardius zonarius barnardi* bill. Photograph taken by A.E.H. at Gluepot Reserve, South Australia.

We observed *B. zonarius barnardi* feeding on the nectar of *E. maculata* flowers from September to November 2022 on Gluepot Reserve in South Australia. Gluepot is characterized by a mallee ecosystem, and *B. zonarius barnardi* is a common species on the property. To feed on the nectar of *E. maculata* flowers, a *B. zonarius barnardi* individual would first pluck a single flower off of the bush with the bill. With the flower in the bill, the individual would then use their bill tips and tongue to orient the flower in their mouth (Figure 1). The flower was always positioned such that it was braced against the inside of the upper bill (Figure 1). With the flower in a secure position, the bird would use the tip of the lower bill to pierce the bulbous base of the floral corolla (Figures 1 and 2), where the nectary is located and the nectar is produced (A.E.H. personal observation). Once the base of the corolla had been pierced, the individual repeatedly protruded and retracted the tongue, contacting the point of incision to access the nectar (Figure 3). When all of the accessible nectar had been consumed, the individual would open the bill and drop the flower on the ground. In all of our observations, the flowers that were fed on and dropped never had remaining nectar inside. This process would be repeated multiple times, with a single *B. zonarius barnardi* individual spending upwards of 10 min at a single *E. maculata* bush, removing flowers and draining them of nectar. The only deviation from this behavior that was observed was when some flowers were picked and oriented in the bill, they were quickly dropped after a single protraction of the tongue. Presumably, these are flowers that did not have any nectar inside, which was determined after a single lick did not result in any nectar capture. We observed this behavior throughout the day and did not observe aggression between *B. zonarius barnardi* individuals and other nectar‐feeding species.

### 2.2 Relevant Morphology

**FIGURE 4:**
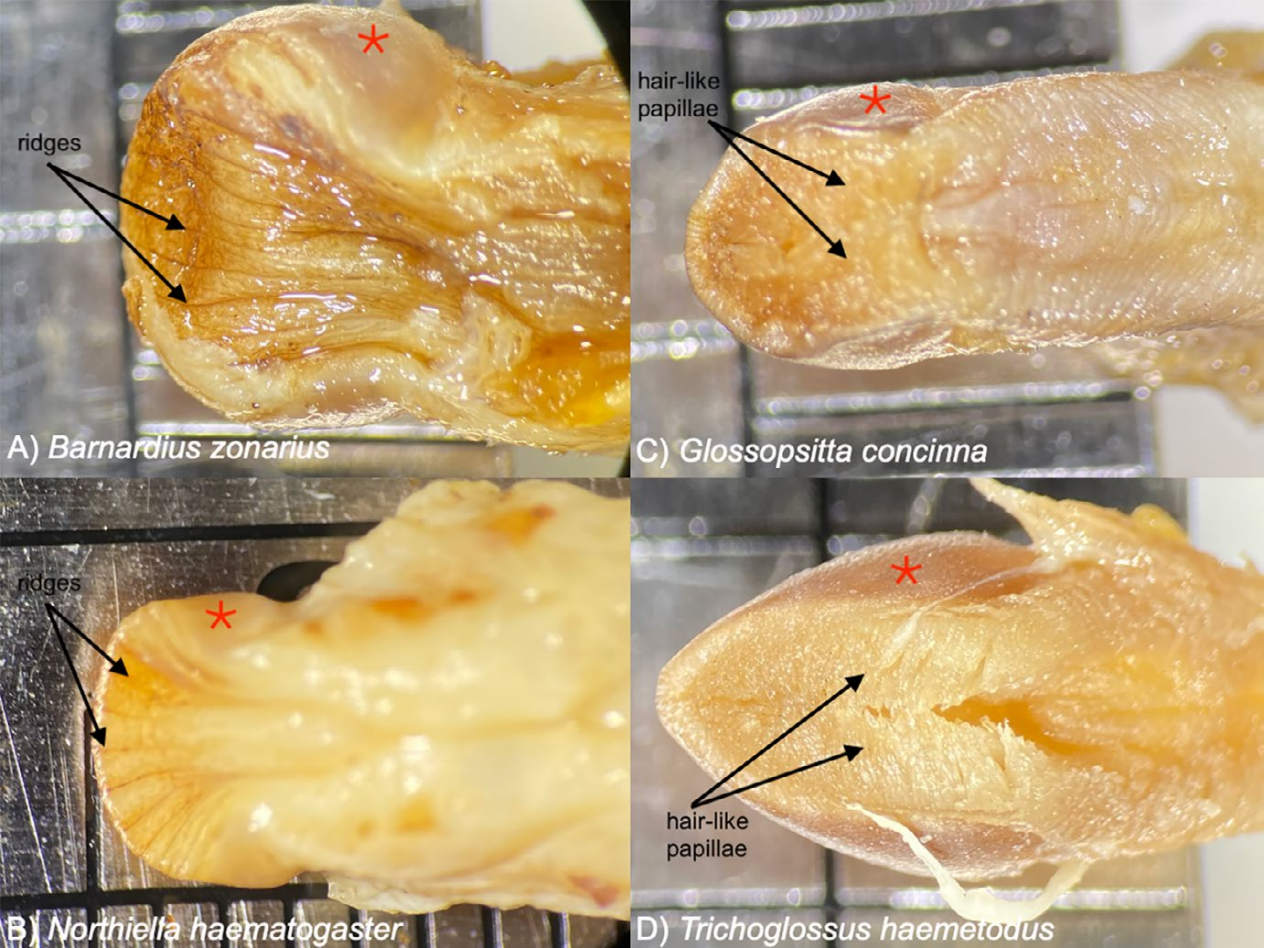
Tongues of parrots illustrating the differences between granivores and nectarivores. Arrows show important features of tongue tip. Red asterisk shows lingual nail, a ubiquitous feature of parrot tongues. Notches on scale bar in each photograph denote 1 mm increments. Photographs taken by A.E.H. at the Queensland Museum, Hendra, QLD. Specimen numbers are: (A) O.32650, (B) O.33441, (C) O.33235, (D) O.33059.

The nectar‐feeding behavior of *B. zonarius barnardi* is notably very different from that seen in lorikeets, and the morphology of the tongue likely plays a role in how the different groups capture nectar (Figure 4). Lorikeets have a distinctive brush‐tipped tongue that they use to feed on floral nectar (Figure 4C,D) and consume pollen (Churchill and Christensen 1970; Smith 1975). While we lack a biomechanical analysis of how lorikeets use their brush‐tipped tongue to capture nectar, we know that they feed by opening their bill around a flower and repeatedly inserting and retracting the tongue, such that the nectar capture is accomplished by the tongue alone and does not involve the bill (Rico‐Guevara unpublished data). Lorikeets so do not typically consume whole flowers or remove flowers from plants when feeding (Rico‐Guevara unpublished data), potentially leaving flowers to replenish.

To understand how *B. zonarius barnardi* consumes nectar, it is helpful to consider the morphology of the feeding apparatus (bill and tongue) and whether they possess any features that could be beneficial for nectar capture, such as the brush‐tipped tongue of the lorikeets (Figure 4C,D). Bill morphology of the genus *Barnardius zonarius* was described by Holyoak (1973) as having deep grooves on the interior ventral surface of the upper bill. These grooves are common in granivorous parrots and are thought to increase the friction of the ventral surface of the upper bill, against which individuals brace seeds when crushing and consuming them (Holyoak 1973). While it is not clear which subspecies of *Barnardius zonarius* was examined, it is likely that these bill ridges are found in all subspecies, as they were also found in other genera of core Platycercini parrots such as *Platycercus* and *Psephotus* (Holyoak 1973). These grooves could be useful for facilitating the nectar‐feeding behavior we observed in a similar way to how they facilitate seed‐eating. *B. zonarius barnardi* holds the flower against the interior ventral surface of the upper bill while extracting nectar with the tongue, and as such these grooves could provide enhanced friction and gripping on that surface, as they are proposed to do when consuming seeds. *Barnardius zonarius* is also noted as having a tongue with no clear modification for any particular feeding strategy, only possessing some shallow grooves at the tip (Holyoak 1973), which matches the specimen we examined (Figure 4A) and is also seen in other closely related species of core Platycercini parrots, such as *Northiella haematogaster* (Figure 4B), *Platycercus*, and *Psephotus* (Holyoak 1973). We did not observe the tongue being inserted into the flower to collect nectar, we only saw the tongue being repeatedly tapped on the bottom of the flower where the nectary is located (Figure 2), and where the corolla was pierced by the lower bill (Figure 3). As such, it is likely that the tongue was being coated with small aliquots of nectar upon contact with the floral base; it is possible that the small grooves in the tongue tip (Figure 4A) could facilitate the coating of the tongue tip in nectar by providing small channels in which capillary action could act to pull nectar on to the tongue, but that remains to be investigated. It is not clear why *B. zonarius barnardi* use this time‐consuming, precise technique to extract nectar, rather than just consuming whole flowers. Flower damage during feeding on *E. maculata* has been observed in *B. zonarius barnardi* previously (Paton and Ford 1977), but the behavior and method of accessing nectar were not described. It is possible that *E. maculata* flowers are too large for *B. zonarius barnardi* to consume whole or that they are distasteful, warranting further investigation.

## 3 Ecological Implications of Nectar Feeding in *Barnardius zonarius barnardi*

### 3.1 Choosing Nectar Resources

During the time when we observed nectar feeding in *B. zonarius barnardi* , the flowering of *E. maculata* was particularly prodigious as South Australia was experiencing rainfall 125% above average, its highest spring rainfall since 2010 (Australian Bureau of Meteorology, December 2022). Honeyeaters are known to track floral resources (Chan 2001) while *Barnardius zonarius* is considered a largely sedentary omnivore that consumes flowers opportunistically (Forshaw 1973; Juniper and Parr 1998; Higgins 1999), with little information available on the degree of flower visitation for specific subspecies. We never observed *B. zonarius barnardi* visiting flowers other than those of *E. maculata* , even though other plants were flowering (e.g., *Grevillea huegelii*, *Myoporum* sp., and other species of *Eremophila*). We do not know how *B. zonarius barnardi* determined which flowers to feed at or why it was not observed feeding at anything except *E. maculata* . It is possible that the high levels of honeyeater activity at *E. maculata* combined with its density across the landscape allowed *B. zonarius barnardi* to cue into it as a food resource through observational learning, which is known to be used by hummingbirds when encountering new resources (Alsthuler and Nunn 2001; Lara, González, and Hudson 2009). While the ways in which nectarivores like hummingbirds and honeyeaters choose nectar resources have been the topic of intense interest (e.g., Pyke 1978, 1981; Alsthuler and Nunn 2001; Burke and Fulham 2003), the mechanisms by which opportunistic nectarivores assess and act on the availability of nectar resources is not well understood.

### 3.2 Potential to Disrupt Interactions With Honeyeaters and Plants

We define opportunistic nectar feeders as species with no morphological or physiological adaptations to nectarivory and who only consume nectar in situations when the resource is highly abundant in the environment or prior to specific life history events (e.g., migration). Opportunistic nectarivory has been recorded in many birds across the globe (e.g., Fleming, Hofmeyr, and Nicolson 2007; Cecere et al. 2011; Machado‐Stredel et al. 2019), but it is particularly interesting in areas where there are also specialized nectarivores, as there arises the potential for competition between species that depend heavily on nectar for survival and those that are attempting to reap the benefits of an easily accessible resource. In the context of Australian ecosystems, honeyeaters are heavily dependent on nectar and are known to compete interspecifically for nectar resources, with larger‐bodied species excluding smaller species from richer resource areas (Ford 1979; Ford and Paton 1982; McFarland 1986). Competition between honeyeaters and other nectarivores for nectar resources and the contribution of opportunistic nectar feeders to the drawdown of communal nectar resources are not well studied (e.g., Dillon 1959; Brooker, Braithwaite, and Estbergs 1990). The potential impact of an opportunistic nectarivore like *B. zonarius barnardi* on this system is interesting, because an individual *B. zonarius barnardi* can sit in an *E. maculata* bush and consume numerous flowers, and due to the nature of their nectar‐feeding strategy, these flowers are removed from the resource pool. When honeyeaters feed, they typically insert the bill into the flower to access nectar and they do not damage the flower in the process, leaving these flowers in the resource pool to continue generating nectar (but short‐billed honeyeaters such as *Melithreptus* sp. will pierce the base of long corollas to access nectar, Paton 1986). If *B. zonarius barnardi* numbers are high in a particular *E. maculata* patch that is also occupied by honeyeaters, as was observed at Gluepot Reserve, then *B. zonarius barnardi* could pose a competition threat by consuming high numbers of flowers and reducing resource availability. Honeyeaters are key pollinators for many Australian plants (Paton and Ford 1977), while *B. zonarius barnardi* appears to be engaging in a form of destructive nectar‐robbing akin to florivory, destroying flowers without offering any pollination services in return. Additionally, because *B. zonarius barnardi* removes flowers from the plant to feed on them, it is reducing the number of fruits that plant can produce and the number of seeds that plant can sire by reducing its available pollen. Many species of *Eremophila* depend on animals for seed dispersal (Richmond and Chinnock 1994) and *E. maculata* seeds in particular are dispersed in emu droppings (Davies 1976).

### 3.3 Extent of Nectar Feeding in *B. zonarius barnardi*

Many bird guides note that *Barnardius zonarius* consumes flowers (Forshaw 1973; Higgins 1999; Menkhorst et al. 2017), but there is often little information provided about which subspecies of *Barnardius zonarius* it is and what species of plant they are visiting (but see Long 1984). Further investigation is needed to determine whether these visits are equally common across all subspecies of *Barnardius zonarius* and whether these visits are targeting nectar consumption, as seen at *E. maculata* , or pollen consumption. As we have seen that *B. zonarius barnardi* will visit flowers explicitly to consume nectar only, it would also be interesting to investigate whether *B. zonarius barnardi* alters their nectar‐feeding behavior based on floral morphology. While we could not find other accounts of *B. zonarius barnardi* feeding on nectar at flowers, there were several accounts of the highly granivorous mulga parrot, *Psephotellus varius*, a close relative of *Barnardius*, opportunistically visiting flowers of *Amyema miquelli* and *Eucalyptus gracilis* (Higgins 1999), and *Grevillea magnifica* (Brown et al. 1997). The impact that *B. zonarius barnardi* has on pollination and interspecific pollinator competition for food resources could differ by plant species depending on the degree to which floral morphology modulates their feeding behavior. For example, if *B. zonarius barnardi* also consumes the nectar from open, cup‐shaped *Eucalyptus* flowers that can be accessed without the destruction of the flower, then *B. zonarius barnardi* could function as a pollinator if it brushes against anthers and stigmas during visits and the flowers remain in the resource pool to be visited by other avian nectarivores. Conversely, if *B. zonarius barnardi* also consumes the nectar from the small, tightly packed flowers of plant genera such as *Amyema* and *Grevillea* where flowers likely need to be removed and consumed, then *B. zonarius barnardi* would play a similar role in the system as with *E. maculata* , removing flowers from the resource pool such that they cannot be used by other avian nectarivores.

## 4 Conclusion

While there has been considerable scientific interest in how honeyeaters interact with plants throughout Australia, the role of opportunistic nectarivores has been relatively overlooked. *B. zonarius barnardi* is likely just one of many opportunistic nectarivores that have heretofore gone undescribed. Understanding how opportunistic nectarivores decide where and when to feed at flowers, how they interact competitively with other nectarivores, and how they alter the resource pool, are all important avenues for future study. Given the unpredictable nature of flowering and nectar availability across the landscape and how that can compound with the inherent climatic variability across the Australian continent, opportunistic nectarivores could play more important ecological roles in Australia compared to areas such as the Neotropics, where resources and flowering are more consistent year to year.

## Author Contributions

**Amanda E. Hewes:** conceptualization (lead), investigation (lead), project administration (equal), resources (equal), writing – original draft (lead), writing – review and editing (equal). **Alejandro Rico‐Guevara:** conceptualization (equal), project administration (equal), supervision (equal), writing – review and editing (equal). **Todd J. McWhorter:** conceptualization (equal), investigation (equal), project administration (equal), resources (equal), supervision (equal), writing – review and editing (equal).

## Conflicts of Interest

The authors declare no conflicts of interest.

## Data Availability

No new data were generated for this work.
